# Production and immobilization of β-glucanase from *Aspergillus niger* with its applications in bioethanol production and biocontrol of phytopathogenic fungi

**DOI:** 10.1038/s41598-021-00237-2

**Published:** 2021-10-25

**Authors:** Hamed M. El-Shora, Reyad M. El-Sharkawy, Aiah M. Khateb, Doaa B. Darwish

**Affiliations:** 1 Department of Botany, Faculty of Science, Mansoura University, Mansoura, Egypt; 2 Botany and Microbiology Department, Faculty of Science, Benha University, Benha, Egypt; 3 Department of Medical Laboratory Technology, College of Applied Medical Sciences, Taibah University, Medina, Saudi Arabia

**Keywords:** Biotechnology, Microbiology

## Abstract

β-Glucanase has received great attention in recent years regarding their potential biotechnological applications and antifungal activities. Herein, the specific objectives of the present study were to purify, characterize and immobilize β-glucanase from *Aspergillus niger* using covalent binding and cross linking techniques. The evaluation of β-glucanase in hydrolysis of different lignocellulosic wastes with subsequent bioethanol production and its capability in biocontrol of pathogenic fungi was investigated. Upon nutritional bioprocessing, β-glucanase production from *A. niger* EG-RE (MW390925.1) preferred ammonium nitrate and CMC as the best nitrogen and carbon sources, respectively. The soluble enzyme was purified by (NH_4_)_2_SO_4_, DEAE-Cellulose and Sephadex G_200_ with 10.33-fold and specific activity of 379.1 U/mg protein. Tyrosyl, sulfhydryl, tryptophanyl and arginyl were essential residues for enzyme catalysis. The purified β-glucanase was immobilized on carrageenan and chitosan with appreciable yield. However, the cross-linked enzyme exhibited superior activity along with remarkable improved thermostability and operational stability. Remarkably, the application of the above biocatalyst proved to be a promising candidate in liberating the associate lignocellulosic reducing sugars, which was utilized for ethanol production by *Saccharomyces cerevisiae*. The purified β-glucanase revealed an inhibitory effect on the growth of two tested phytopathogens *Fusarium oxysporum* and *Penicillium digitatum*.

## Introduction

β-Glucan is the third abundant hemicelluloses present in nature. It is generally composed of β-(1,3)-glucan with β-(1,6)-glucan branches. Interestingly, β-glucan is predominantly distributed amongst different plants and fungi with pathophysiological interest^1–6^. β-glucanase (EC 3.2.1.6) belongs to glycosyl hydrolase family that hydrolyzes β-glucan polysaccharide, producing 3-*O*-cellotriosyl-d-glucose and 3-*O*-cellobiosyl-d-glucose ^7^. β-glucanase is also capable of binding to different insoluble and unhydrolyzable polysaccharides. The products of the enzyme action expressed growth elevation of valuable probiotic bacteria^8^ and anti-hypercholesterolemia^9^. Furthermore, β-glucanase has been exploited in production of ethanol^10^. The enzyme is capable of protecting plants against different fungal pathogens^5,8^. Hence, β-glucanase is receiving increasing attention as a promising industrial enzyme.

Enzymes are commonly applied in production of several substances that need excessive cost for their production by chemical processes. So, the optimization of different culture conditions for improving β-glucanase production is of immense interest but the enzymatic processes have unfavorable drawbacks including sensitivity to harsh environmental conditions, difficulties during regeneration as well as recycling processes. Immobilization allows the enzyme to conquer harsh conditions and offer a vital area of study in the field of industrial biotechnology^11–13^. Furthermore, immobilization enables enzymes to work with great catalytic activity and consequently diminish the budget of enzymatic processes^12–15^. Several immobilization techniques can be employed such as encapsulation, covalent-binding, crosslinking, entrapment, and adsorption^12,15–17^. Cross-linking with chitosan and covalent binding using carrageenan are of significant concern^12,16–18^.

*A. niger* has been considered as an important producer of β-glucanase^6,19^. The degradation of different agricultural wastes is of great interest because it assists reusing of these materials and the sensible use for byproducts. Furthermore, the application of different hydrolytic enzymes, particularly β-glucanase, on many lignocellulosic materials is task for production of bioethanol as a supplier of renewable energy^20^. Antifungal proteins are of significant biotechnological interest in biocontrol of various phytopathogenic fungi. The biocontrol potentiality of such proteins is accomplished by degrading fungal cell polymers and inactivating ribosomes, hinting weakens and lysis of fungal cells. *Fusarium oxysporum* triggers wilt disease of tomato as important crop^21^. Also, *Penicillium digitatum* initiates postharvest diseases for citrus fruits and raises the economic loss. So, discovering an alternative way for enhancing plant defence against fungal diseases is essential approach^21–23^.

The agricultural wastes represent an environmental problem since they cause some sort of pollution. Therefore, it was thought to use fungal enzyme to convert them to useful material for production of bioethanol. Hence, the novelty of this work is presented by production of bioethanol and biocontrol of phytopathogenic fungi using purified fungal β-glucanase. The aims of the present work were: firstly, to investigate the optimal culture conditions for β-glucanase production by *Aspergillus niger*. Secondly, to purify and immobilize the enzyme by covalent-binding and cross-linking techniques. Thirdly, to investigate the capability of β-glucanase in degradation of different lignocellulosic wastes, production of bioethanol and biocontrol of pathogenic fungi.

## Results and discussion

### Molecular identification of β-glucanase producing fungus

**Figure 1:**
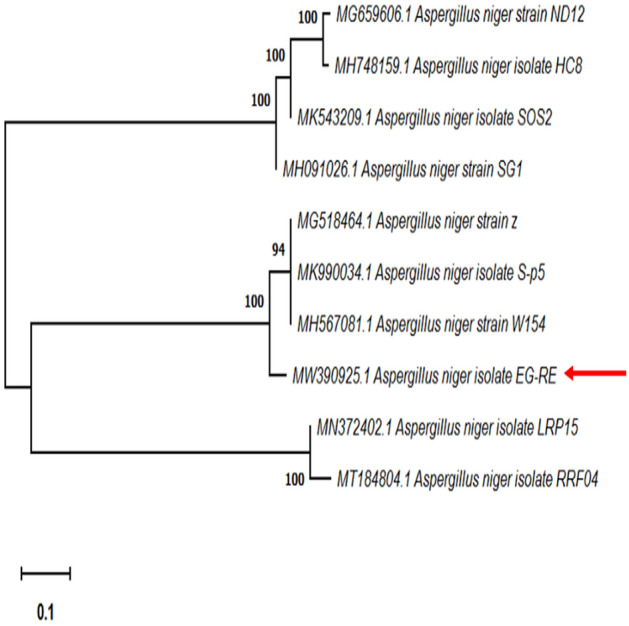
Molecular phylogenetic tree of *A. niger* EG-RE based on ITS sequence of rDNA using MEGA-X software based on the Neighbor-Joining method. The bar length represents 0.1 substitutions per nucleotide site. The isolate in the present study is indicated by red arrow.

*Aspergillus niger* was used as a source of β-glucanase in the present research. The fungal strain was genetically characterized using PCR method and the GenBank accession no. was MW390925.1 (Fig. 1).

### Bioprocess optimization of *A. niger* β-glucanase productivity

**Figure 2:**
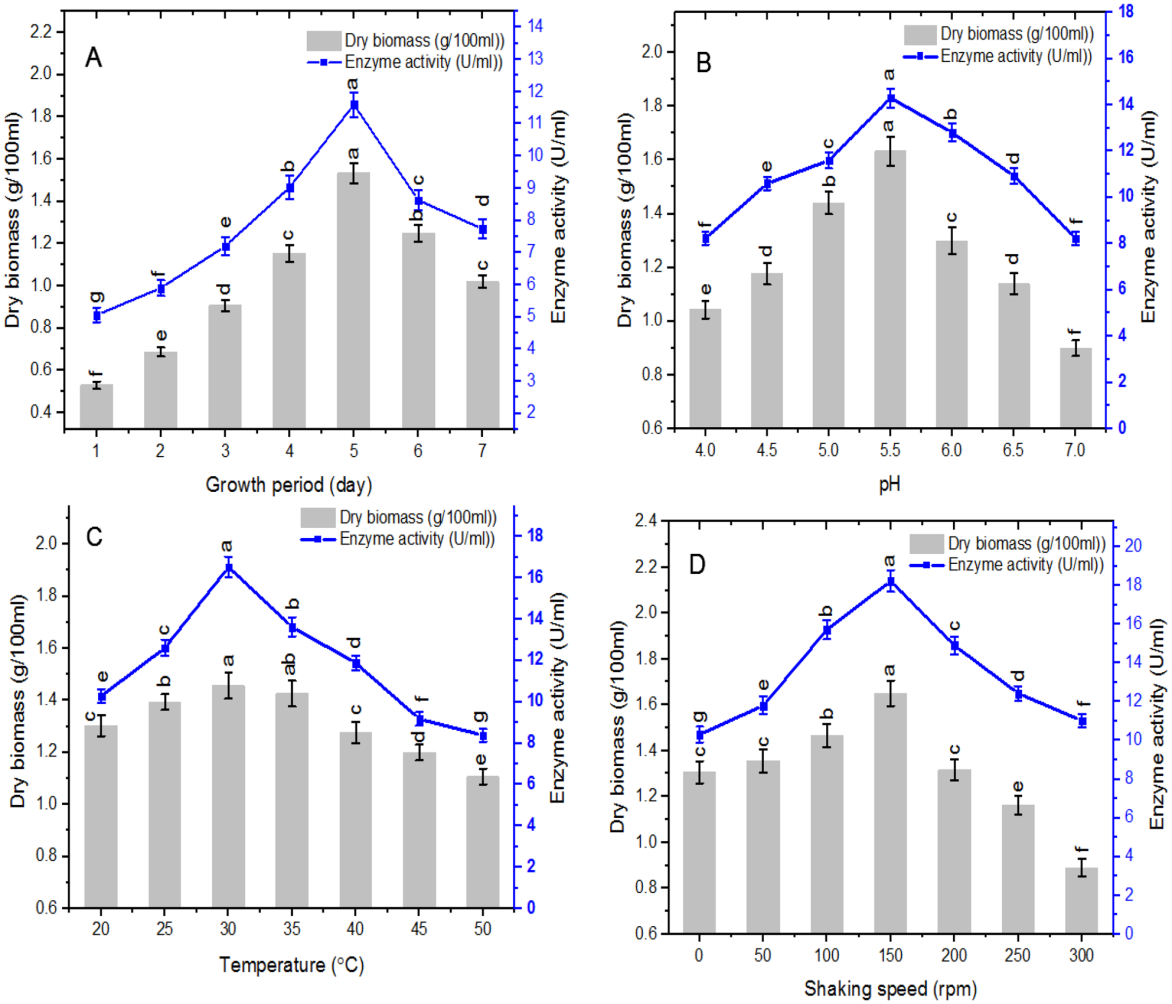
**(A)** Time-course production, **(B)** effect of initial pH, **(C)** effect of temperature, **(D)** effect of agitation on β-glucanase production and dry biomass of *A. niger.* Enzyme activity was assayed at 40 °C using CMC as substrate. Vertical bars were displayed as mean ± standard deviation, various letters represent level of significance at P < 0.05, n = 3.

During the exponential phase, the magnitude of β-glucanase was dramatically increased and subsequently declined during the stationary phase (Fig. 2A). Therefore, the following experiments were operated at the 5th day of incubation period, where the highest level of β-glucanase was established. β-glucanase activity and the dry biomass of *A. niger* were investigated at pH range 4–7 (Fig. 2B). There was an increment in β-glucanase activity up to pH 5.5 after which the activity declined sharply at the higher pH values. The results also reveal that there was continuous increase in the dry biomass of *A. niger* from pH 4.0 to pH 5.5. The dry biomass of mycelium was then reduced progressively at elevated pH values. The results shown in Fig. 2C revealed that β-glucanase activity and dry biomass of *A. niger* displayed continuous increase with increasing the temperature up to 30 °C where the maximum enzyme activity and dry biomass were reached. After 30 °C there was a snappy decrease during the elevation in the reaction temperature. The optimal values of β-glucanase and dry biomass were recorded at 150 rpm and dropped thereafter (Fig. 2D). The results are in accordance with the report for most β-glucanases^4,6,19,24–26^.

**Figure 3:**
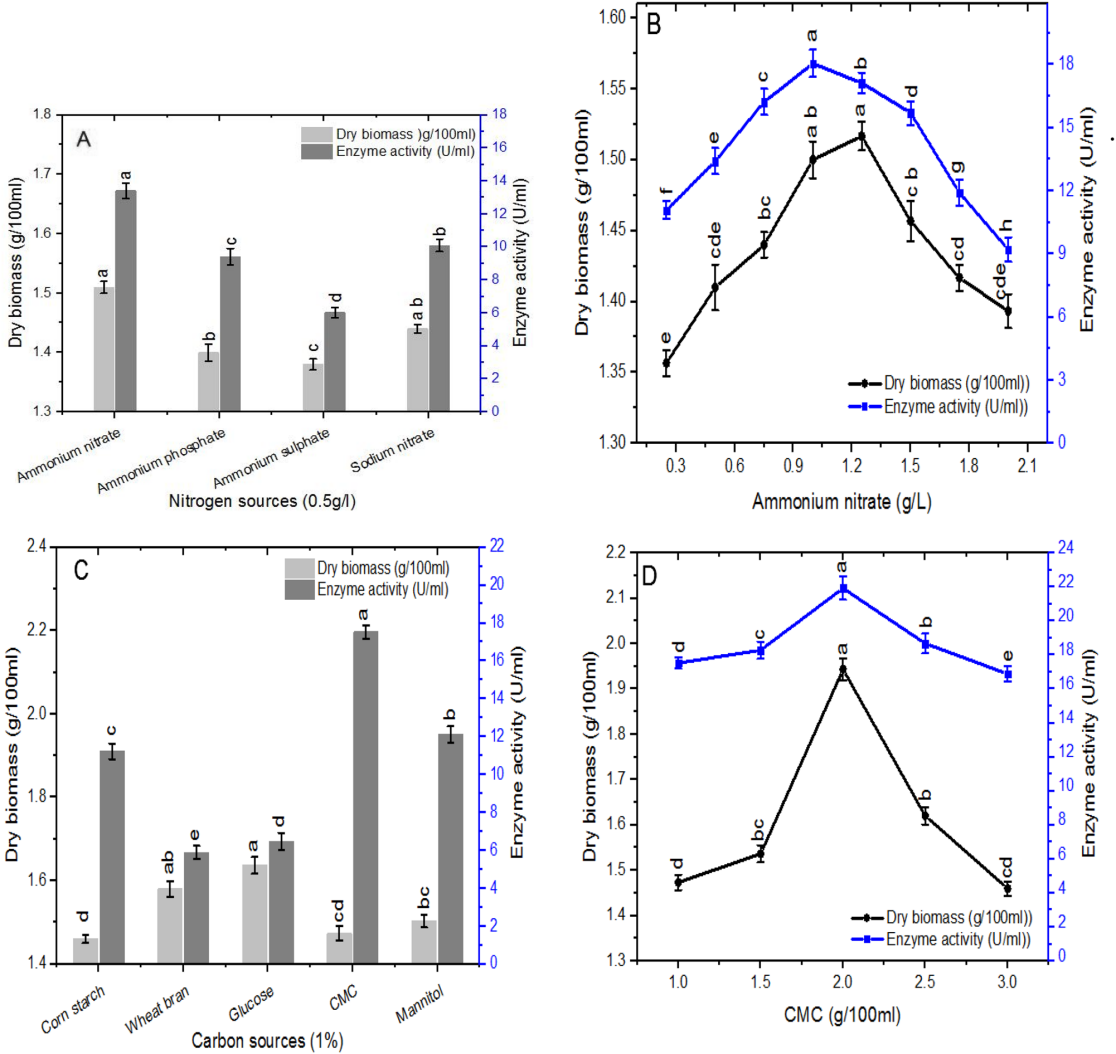
**(A)** Effect of different nitrogen sources, **(B)** effect of various concentrations of ammonium nitrate, **(C)** effect of different carbon sources, **(D)** effect of various concentrations of CMC on β-glucanase production and dry biomass of *A. niger.* Vertical bars were displayed as mean ± standard deviation, various letters represent level of significance at P < 0.05, n = 3.

Screening of four nitrogen sources showed that ammonium nitrate was the best nitrogen source for both enzyme production and dry biomass (Fig. 3A). In contrast, ammonium phosphate and ammonium sulphate were the noblest nitrogen sources for β-glucanase production from *Penicillium oxalicum*^27^. Studying the effect of various concentrations of ammonium nitrate (Fig. 3B) revealed that 1 g/L ammonium nitrate was the best to support the highest enzyme production whereas 1.25 g/L was the best for biomass production. It was reported that ammonium nitrate as nitrogen source supported the maximum activity of β-glucanase from *Trichoderma* sp.^25^.

The results in Fig. 3C reveal that the best carbon source for β-glucanase production was CMC whereas glucose was the best for biomass production. *P. oxalicum* and *P. citrinum* exhibited maximum enzyme activity with methylcellulose as a carbon source^27,28^. Both glucose and lactose as a carbon sources promoted β-glucanase production. The proficiency of β-glucanase to hydrolyze CMC and cellulose was determined^26,29^. The catabolite repression for β-glucanase production by using 1% glucose as carbon source was confirmed^4,29^. The optimal concentration of CMC for the enzyme production was 2% (Fig. 3D) which is in agreement with the results of^29^.

### Purification of *A. niger* β-glucanase

**Figure 4:**
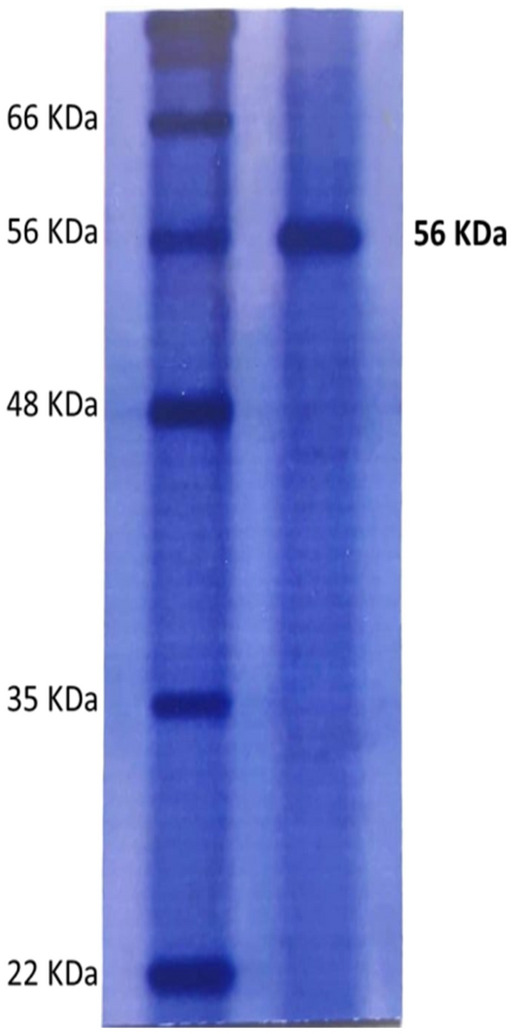
SDS-PAGE of purified *A. niger* β-glucanase. Lanes are assigned as follows: lane M: markers and Lane PE: purified enzyme (uncropped gel image of this figure is in the supplementary information Fig. S1).

The purity of β-glucanase from Sephadex G-200 was about 10.33-fold with a specific activity of 379.1 U/mg protein (Table 1). The purified enzyme has a molecular weight of 56 KDa as detected from SDS-PAGE (Fig. 4, uncropped gel image of purified β-glucanase in Fig. S1). β-glucanase from *A. niger* displayed 42.6% yield and 5.4-fold of purification^30^. β-glucanase from *A. niger* was purified to homogeneity with single protein band of 32 KDa with specific activity of 33.3 Umg^-1^ protein and14.24-fold^31^.

**Table 1:** Purification summary of β-glucanase produced by *Aspergillus niger*.

Purification steps	Total protein (mg)	Total activity (U)	Specific activity U mg^−1^	Fold purification	% yield
Crude extract	166	6093.9	36.7	1	100
Ammonium sulfate precipitation (85%)	32.52	4465.2	137.3	3.74	73.3
Dialysis	12.96	3168.0	244.4	6.66	51.9
DEAE-Cellulose	5.94	1804.5	303.8	8.28	29.6
Sephadex G-200	1.15	436.0	379.1	10.33	7.2

### Detection of amino acid residues of purified β-glucanase

*N*-Acetyl imidazole (NAI), phenylmercuric acetate (PMA), *N*-bromosuccinimide (NBS) and 2.3-butanediol (2,3-BD) are well known reagents for tyrosyl, sulfhydryl, tryptophanyl and arginyl residues, respectively in enzymes protein^12,31,32^. Therefore, these compounds were tested regarding to their effects on β-glucanase activity and the results in Fig. S2 indicated an inhibition of the enzyme activity by these compounds. The IC_50_ of the above four compounds were 8.87, 5.47, 6.44 and 4.65 mM, respectively. These results reveal the essentiality of the four residues for β-glucanase catalysis.

### Immobilization of β-glucanase by two methods

**Figure 5:**
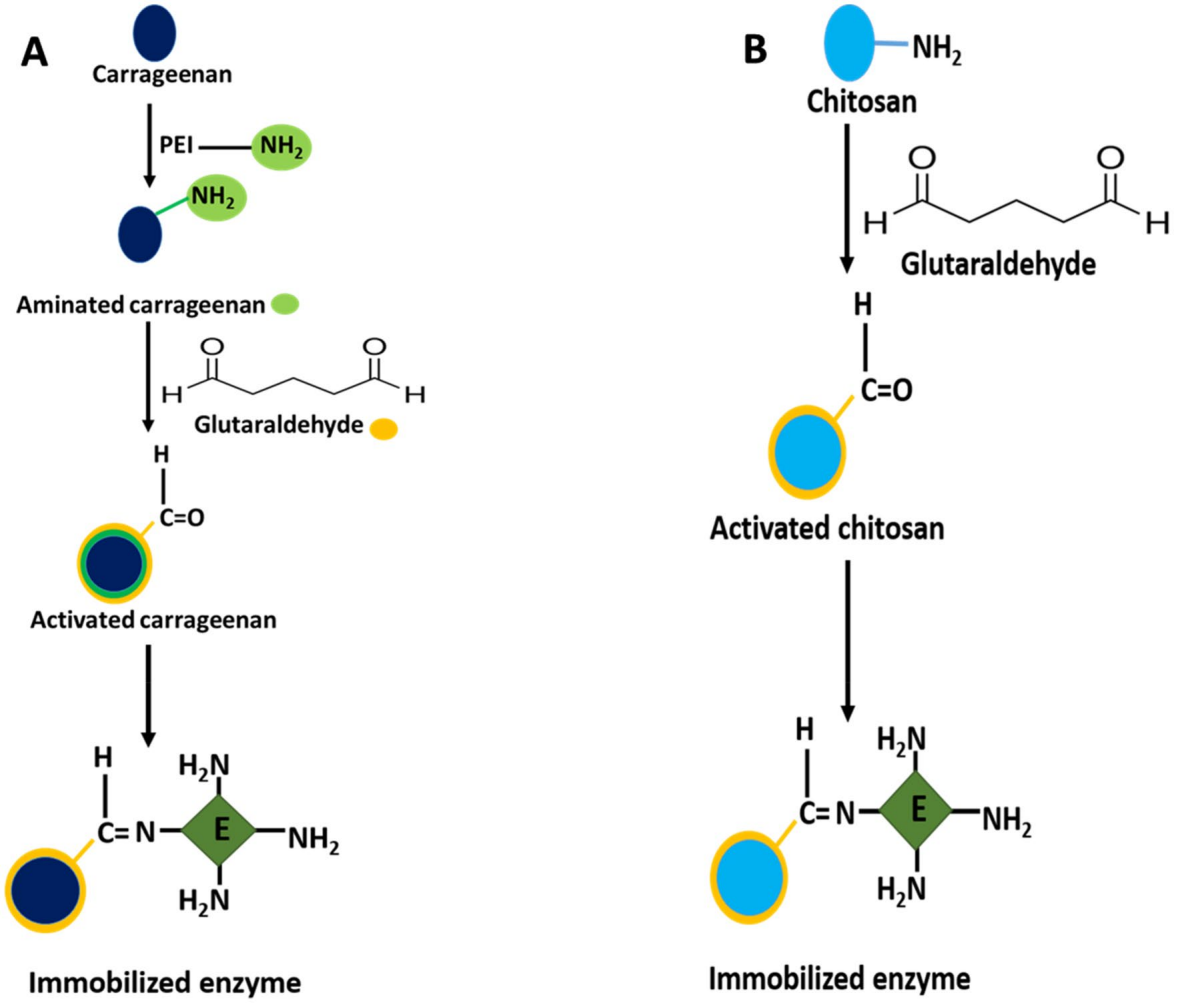
Schematic representation of β-glucanase immobilization on activated carrageenan **(A)** and chitosan **(B)** beads.

β-Glucanase from *A. niger* was immobilized by covalent binding using carrageenan and cross-linking using chitosan. The schematic diagram showing the immobilization process of β-glucanase was illustrated in Fig. 5. The yield of immobilization was 70.3% and 81.3% with carrageenan and chitosan, respectively (Fig. S3). The immobilization of enzyme led to an increase in enzyme rigidity, which is generally reflected by improving the stability against denaturation on rising the temperature^12,33^. The successful immobilization of β-glucanase on carrageenan and chitosan was reported by various investigators^8,16,34^.

The cross-linked β-glucanase was initially increased using low concentration of glutaraldehyde. Application of more than 20% v/v glutaraldehyde severely reduced the activity of the enzyme (Fig. S4). The reduction in the enzyme activity might be attributed to the interaction between lysine residues at the catalytic site of β-glucanase with the glutaraldehyde-aldehyde groups. This endorses cross-linking of the protein chains, hindering the active site of the enzyme and finally causes enzyme inactivation during the stabilization process^8^.

### Characterization of free, covalent-bound and cross-linked β-glucanases

There was a little fluctuation in the immobilized β-glucanase activity and stability among different temperatures comparing with the free enzyme. The optimal temperatures were respectively found to be 45 °C, 50 °C and 55 °C for the free, covalent-immobilized and cross-linked enzyme (Fig. S5A). The thermal stability at 70 °C of the cross-linked enzyme was higher than the covalent-immobilized enzyme (Fig. S5B). This effect may be due to the protecting effect of immobilization process at the elevated temperatures at which deactivation of the enzyme takes place. Thermostability of the enzyme depends on its microenvironment and its subunit re-cognization. Furthermore, immense repulsions offer confirmation and stability for the proteins. The repulsion between charged groups sited in the protein is the major driving force for stability of the enzyme^8,12,32^.

**Figure 6:**
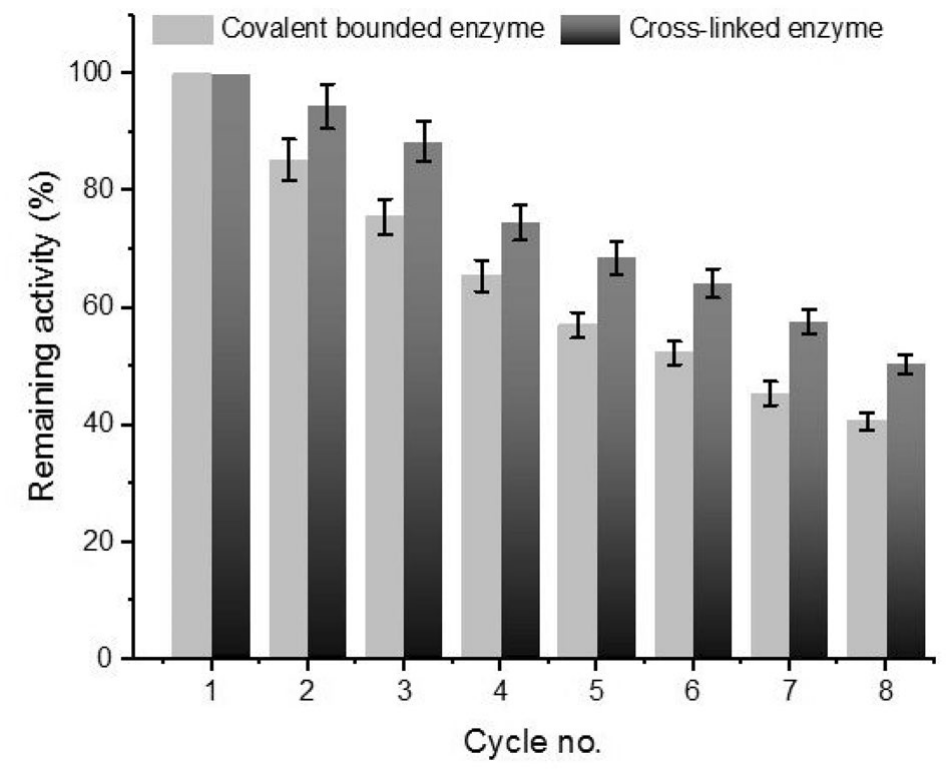
Reuse of immobilized β-glucanase from *A. niger*. Enzyme activity was assayed at 40 °C using CMC as substrate. Vertical bars were displayed as mean ± standard deviation.

The reuse of immobilized β-glucanase was investigated through 8 sequential cycles (Fig. 6). At the optimal conditions, the remained activity of cross-linked β-glucanase was better than that of carrageenan-immobilized enzyme throughout 8 cycles. The reduction of β-glucanase activity throughout the 8 cycles might be ascribed to protein damage, protein deactivation, and physical damage of the immobilized beads-bound protein^13,35^.

### Effect of β-glucanase on wheat bran and saw dust pretreated with acid and alkali

**Figure 7:**
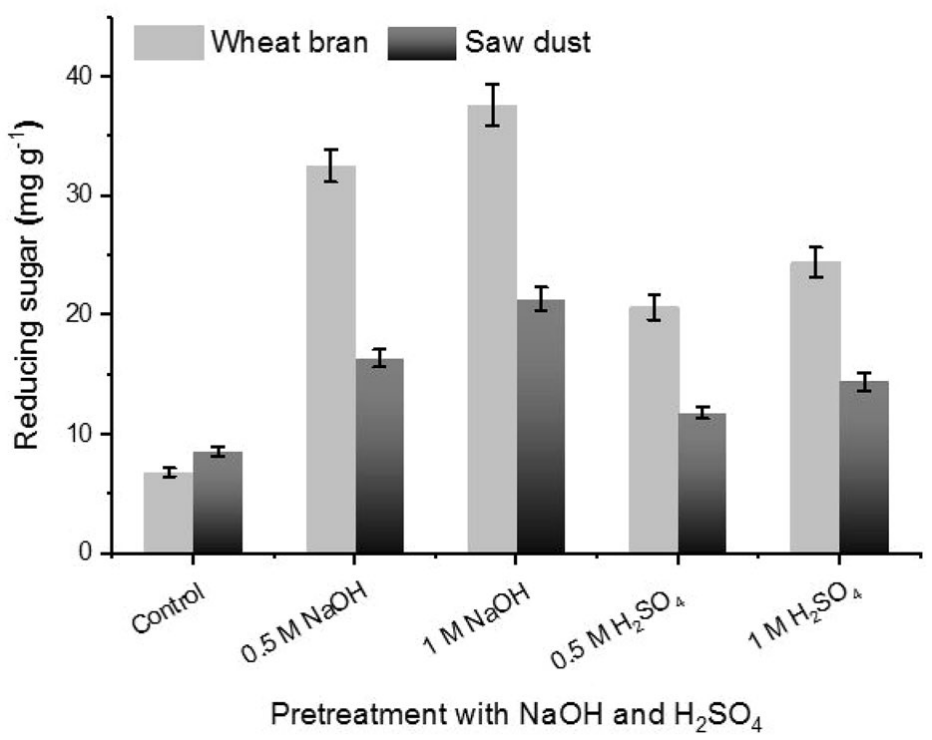
Effect of β-glucanase on wheat bran and saw dust pretreated with H_2_SO_4_ or NaOH. Control sample was carried out with distilled water. Enzyme activity was assayed at 40 °C using CMC as substrate. Vertical bars were displayed as mean ± standard deviation.

The influence of β-glucanase on oven dried-wheat bran and sawdust pretreated with H_2_SO_4_ and NaOH is illustrated graphically in Fig. 7. The pretreatment of wheat bran and poplar sawdust with different concentrations of H_2_SO_4_ and NaOH enhanced production of reducing sugars when compared with untreated (the control). The produced amount of reducing sugar from wheat bran and sawdust of poplar was higher after pretreatment with NaOH than H_2_SO_4_. In support, it has been reported that acidic pretreatment were less effective than alkaline pretreatments^36–39^.

### Bioethanol production

**Figure 8:**
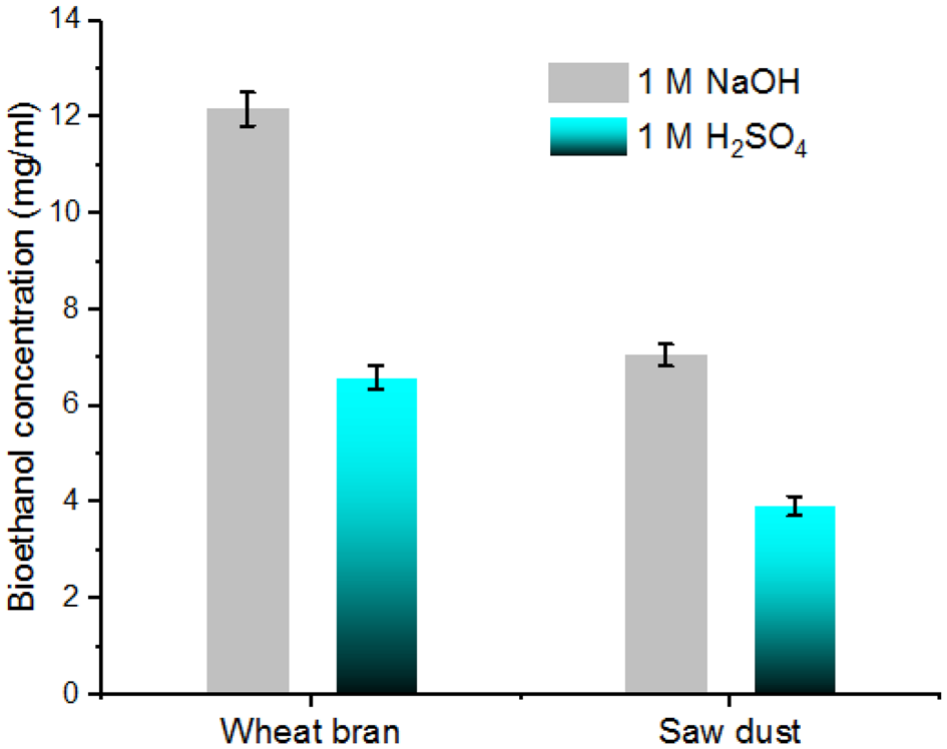
Bioethanol production from the fermentation of wheat bran and sawdust pretreated with 1 M NaOH or 1 M H_2_SO_4_. Vertical bars were displayed as mean ± standard deviation.

After acid or alkali hydrolysis, the produced sugars were fermented by *Saccharomyces cerevisiae* to bioethanol. Figure 8 clearly illustrates that the bioethanol production from wheat bran was higher than that from sawdust after fermentation process. Similar results have been described by other researchers^37,39–41^. The enhancement of bioethanol production from various lignocellulosic sources, particularly agricultural wastes, is attained by adopting different hydrolytic enzymes including cellulases as well as hemicellulases^38–41^. Lignocellulosic wastes are produced widely throughout the world particularly wheat bran and sawdust from agricultural industries and they are consumed for production of renewable fuels.

### Biocontrol activity of β-glucanase

Biocontrol of phytopathogenic fungi by various hydrolytic enzymes is a favorable nonchemical policy for crop protection. Thus, the antifungal capability of purified β-glucanase against *F. oxysporum* and *P. digitatum* was investigated (Fig. S6). *F. oxysporum* and *P. digitatum* seemed to be significantly sensitive to β-glucanase compared to the control plates. The antagonistic effect of purified fungal enzymes was approved by^21,42–45^. Biocontrol of various fungal pathogens by β-glucanase produced from *Trichoderma harzianum* attributed to its hydrolytic capability to degrade β-glucan and accordingly damage the fungal cell wall^44,46^. Several researchers proved the antifungal and anticancer activities of different bioactive fungal-derived materials^21,44–46^. In addition, previous investigation revealed that β-glucanase has an inhibitory effect on the pigmentation as well as the cell walls of certain molds such as *Rhizoctonia solani* and *Pythium aphanidermatum.* However, *Fusarium oxysporum* was resistant and this supports the dissimilarity in the polysaccharides content and composition of fungal cells^44^. The differences in the hydrolytic capability of β-glucanase toward different fungal cell walls may be attributed to the presence of unique amounts and types of different polysaccharides^43,44,47^.

## Material and methods

The overall processes of fungal isolation, molecular identification of the potent fungal isolate, bioprocess optimization of β-glucanase production, enzyme purification, immobilization and biotechnological applications are illustrated in Fig. S7.

### Molecular identification of the fungal strain producing β-glucanase

Genomic DNA, which was extracted from *A. niger* EG-RE, was applied as a template for PCR amplification. The primer ITS-1 (5′–TCC GTA GGT GAA CCT GCG G-3') and the primer ITS-4 (5'-TCC TCC GCT TAT TGA TAT GC-3') were utilized to amplify the nuclear ribosomal ITS-rDNA region. The sequencing results were submitted to the GenBank database for sequence alignment using the Basic Local Alignment Search (BLASTn) Tool (http://www.ncbi.nlm.nih.gov/Blast). Phylogenetic tree and molecular evolutionary analysis was conducted by the Neighbor-Joining method^48^.

### Preparation of enzyme extract

*A. niger* (10^6^ spores/ml) was grown on CMC medium (carboxymethyl cellulose, pH 5.5) containing (g/L): 20.0 CMC, 1.0 NH_4_NO_3_, 5.0 yeast extract, 1.0 K_2_HPO_4_, 1.0 KCl, 0.01 FeSO_4_.7H_2_O and 0.5 MgSO_4_.7H_2_O. Flasks were then incubated in complete darkness at 25 °C for 5 days with a shaking speed of 130 rpm. After filtration, the mycelium fragments were eliminated through centrifugation at 4 °C for 10 min at 10,000 rpm. The resulting supernatant signified crude β-glucanase and then stored at − 20 °C for future use^17^.

### Assays of β-glucanase and protein content

The enzyme activity was determined as described by^27^ through estimation of produced reducing sugar in fermentation medium. The content of reducing sugar was evaluated colorimetrically using DNS (3,5-dinitrosalicylic acid). The reaction medium composed of 0.5 ml of 0.5% CMC, 2 ml citrate buffer (50 mM, pH 5.0) and 0.5 ml of enzyme preparation. The mixture was kept for 20 min in water bath at 40˚C. The reaction was stopped by 3 ml of DNS reagent. The absorbance was recorded with a spectrometer at 540 nm. One unit (U) of β-glucanase activity was expressed as μmol glucose formed per min under normal assay conditions. The protein concentration was assayed according to^49^, using bovine serum albumin as standard.

### Optimization of fungal growth and β-glucanase production

For the bioprocess optimization of *A. niger* β-glucanase productivity, the enzyme activity and the associated biomass of *A. niger* were monitored at different growth phases. The influence of different pH was investigated within the range of 4.0–7.0. The impact of temperature was determined by incubating flasks in the dark at varied temperatures (20, 25, 30, 35, 40, 45 and 50 °C). The impact of agitation was examined by incubating the culture with numerous agitation speeds (50, 100, 150, 200, 250 and 300 rpm). Different nitrogen sources (sodium nitrate, ammonium nitrate, ammonium sulphate and ammonium phosphate) at 0.5 g/L and different carbon sources (corn starch, wheat bran, glucose, CMC, and mannitol) at 1% w/v were tested individually for their effect on enzyme production. The enzyme productivity was monitored at various ammonium nitrate concentrations (0.25, 0.50, 0.75, 1.0, 1.25, 1.50, 1.75 and 2.0 g/L) and different concentrations of CMC in medium (0.5, 1.0, 1.5, 2.0, 2.5 and 3.0 g/100 ml).

### β-Glucanase purification

Crude preparation of β-glucanase was subjected to ammonium sulfate to achieve 85% saturation and kept at 4 °C with soft stirring for 24 h. The content was then centrifuged for 20 min at 8000 rpm. The pellets were dissolved in 1 mM potassium phosphate buffer (50 ml, pH 5.5) and afterwards desalted by dialyzing for 24 h against the same buffer. The dialyzed suspension was then applied onto equilibrated DEAE-Cellulose column (2 cm × 120 cm). The bound protein is eluted with the same buffer. At a flow rate of 3 ml/min, fractions with β-glucanase activity were eluted. The active fractions were pooled, dialyzed and subjected to Sephadex G200 column pre-equilibrated using the same buffer (1 mM, pH 5.5). The eluted fractions were then monitored spectrophotometrically at 540 nm and the fractions rich with β-glucanase activity were stored at 4 °C for enzyme characterization^32,43^. The molecular weight and purity of β-glucanase were carried out as described by^50^ using SDS-PAGE.

### The active groups of β-glucanase

The reagents of active groups comprising *N*-acetylimidazole (NAI), *N*-bromosuccinimide (NBS) phenylmercuric acetate (PMA) and 2.3-butanediol (2,3-BD) on β-glucanase activity were explored. β-glucanase activity was expressed as the remaining activity of the control.

### Immobilization of β-glucanase on carrageenan and chitosan beads

The purified β-glucanase was immobilized on κ-carrageenan as activated bead by 20% glutaraldehyde^51^. The purified β-glucanase was immobilized on chitosan beads-activated with 20% glutaraldehyde. The immobilization efficiency of β-glucanase was examined using different concentrations of glutaraldehyde (4, 8, 12, 16, 20 and 24% v/v)^17,52^. The immobilized enzyme (0.1 g) was used for β-glucanase assay as designated earlier for the free enzyme.

### Optimum temperature and thermostability of free and immobilized β-glucanase

The reaction was performed for 40 min at various temperatures (20, 25, 30, 35, 40, 45, 50, 55 and 60 °C) followed by enzyme determination^12^. Thermostability of the β-glucanase was examined after preincubating the free and immobilized enzyme at 70 °C without substrate at various time intervals (10–60 min). The residual enzyme activity was measured at each time interval under standard conditions.

### Reuse of immobilized β-glucanase

The activity of immobilized enzyme (covalent bound and cross-linked) was examined for eight cycles followed by measuring the enzyme activity. The relation between the remaining enzyme activity and the cycle number was plotted.

### Effect of β-glucanase on the pretreated lignocellulosic waste

Pretreatment of wheat bran and sawdust was carried out by NaOH (0.5 and 1 M) and H_2_SO_4_ (0.5 and 1 M). In 1L of such solutions, 100 g of waste was mixed and incubated at 72 °C for 1 h. The treated biomass was then filtered, washed several times with distilled water until the washed water become neutral and then dried at 60 °C. Also, control sample was performed with distilled water and considered as control^37,40^.

The enzymatic digestibility was made in a tube containing 0.5 g of the immobilized enzyme, with 2% w/v concentration of the oven dried-pretreated lignocellulosic waste in citrate buffer (0.05 M, pH 5.0), for 4 h at 50 °C. The mixture was then centrifuged and the resulting supernatant was employed in the determination of reducing sugars produced from pre-treated lignocellulosic waste. The samples without enzyme were defined as blank. After treatment of lignocellulosic waste with β-glucanase, the quantity of reducing sugars (mg/g) was determined via DNS method.

### Production of bioethanol by β-glucanase

The acid hydrolysate was neutralized with Ca(CO_3_)_2_ to pH value between 5.0 and 6.0 and washed with distilled water. Both acid and alkali hydrolysate were treated with β-glucanase at 45 °C for 4 h. The fermentation procedure was performed in presence of *Saccharomyces cerevisiae* as described to^40^.

### In vitro antifungal activity of β-glucanase enzyme

The inhibitory effect of β-glucanase was carried out as described by^44,47^. The purified β-glucanase was carried out against two phytopathogenic fungi namely, *F. oxysporum* and *P. digitatum* on Sabouraud Dextrose Agar (SDA) using hyphal growth inhibition assay. The fungal mycelium was put into the wells of SDA having 200 µl of β-glucanase in phosphate buffer (100 mM, pH 6.0). The fungal inhibition was assessed after 7 days incubation. Negative control having the buffer without enzyme was performed.

### Statistical analysis

All experimental results were done in triplicate, and data were taken as mean ± standard deviation (S.d.). The obtained results were analyzed statistically via the Statistical Package for Social Sciences (SPSS) version 25 (IBM, Armonl, Ny, USA) using ANOVA (analysis of variance) supplemented by Tukey´s HSD test at level of significance of P < 0.05. Different letters represent the significant differences between samples.

## Conclusion

In this study, β-glucanase productivity by *A. niger* was maximized through nutritional optimization bioprocess. Ammonium nitrate and CMC was the best nitrogen and carbon sources, respectively. The purified enzyme was immobilized on two different biopolymers; carrageenan and chitosan. Both supporters proved to be operative biomaterials for β-glucanase immobilization. However, the cross-linked enzyme was more efficient compared to covalent immobilized one. The purified β-glucanase greatly released the associated lignocellulosic reducing sugars, which was subsequently employed for ethanol production by *S. cerevisiae*. The purified enzyme from *A. niger* exhibited an inhibitory effect on the growth of two tested phytopathogens *F. oxysporum* and *P. digitatum*. β-glucanase from *A. niger* is depicting a promising candidate of biotechnological interest which provides a broad future perspective in the biodigestibility of different lignocellulosic wastes and also in plant protection against fungal pathogens. Further studies are demanded to find out the mechanism of fugal degradation of cellulosic materials as well as the mechanism of antifungal activity.

## Supplementary Information

Supplementary Figures.

## Data Availability

All data generated and analyzed during this study are included in this article.
