# Supplementary material for: Production and immobilization of β-glucanase from Aspergillus niger with its applications in bioethanol production and biocontrol of phytopathogenic fungi
Source: Sci Rep. 2021 Oct 25;11:21000. doi: 10.1038/s41598-021-00237-2 (PMC8545931; doi:10.1038/s41598-021-00237-2)

**β-glucanase from *Aspergillus niger*: Production, Immobilization and Biotechnological Applications**

**Hamed M. El-Shora^1^*, Reyad M. El-Sharkawy^2^, Aiah M. Khateb^3^, Doaa B. Darwish^1,4^**

^1^Department of Botany, Faculty of Science, Mansoura University, Egypt.

^2^Botany and Microbiology Department, Faculty of Science, Benha University, Egypt.

^3^Department of Medical Laboratory Technology, College of Applied Medical Sciences, Taibah University

^4^Department of Biology, Faculty of Science, University of Tabuk, Tabuk, Saudi Arabia

* **Corresponding author:** [shoraem@yahoo.com](mailto:shoraem@yahoo.com); [shora@mans.edu.eg](mailto:shora@mans.edu.eg)

**Contributed equally to this work**


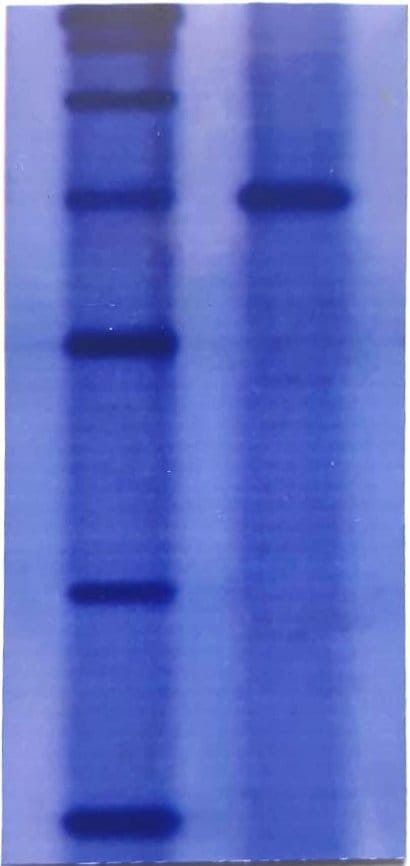


**Purified enzyme**

**Supplementary Figure S1.** **Uncropped gel image of purified β-glucanase produced by *A. niger* in Figure 5.**


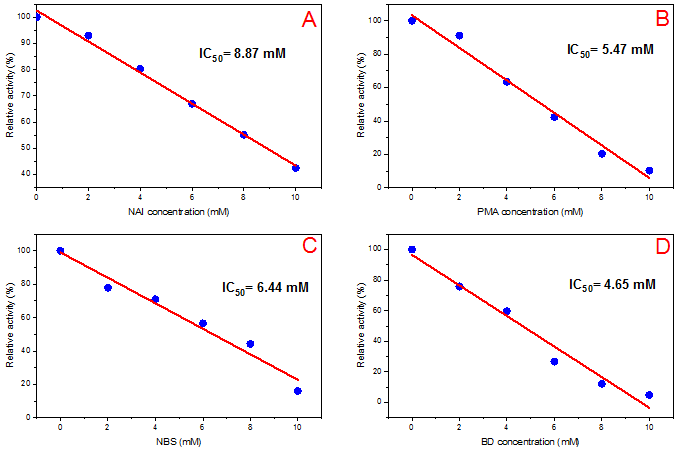


**Supplementary Figure S2. Effect of NAI (A), PMA (B), NBS (C) and 2.3-BD (D) on purified β-glucanase produced by *A. niger.***


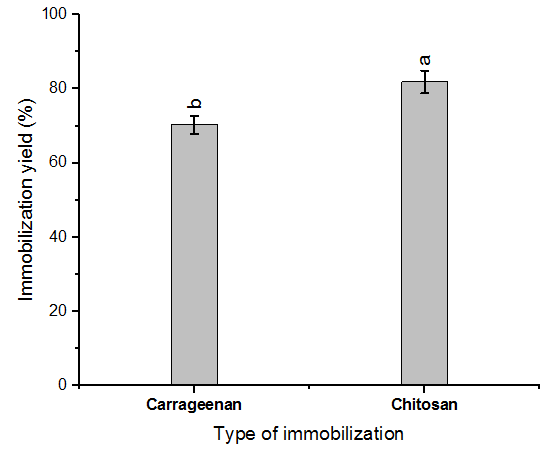


**Supplementary Figure S3. Immobilization of β-glucanase from *A. niger* by covalent binding and cross-linking methods. Enzyme activity was assayed at 40^o^C using CMC as substrate. Vertical bars were displayed as mean ± standard deviation.**


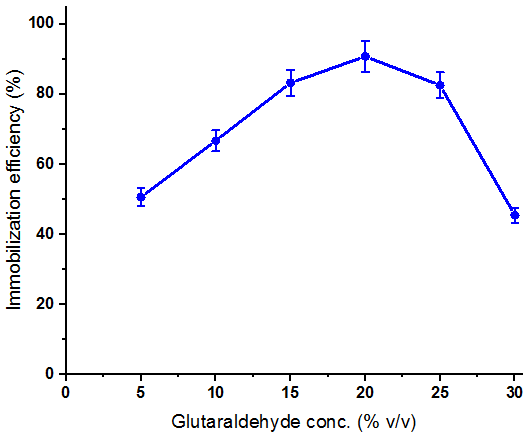


**Supplementary Figure S4. Influence of glutaraldehyde concentrations on immobilization efficiency of β-glucanase from *A. niger* on chitosan.**


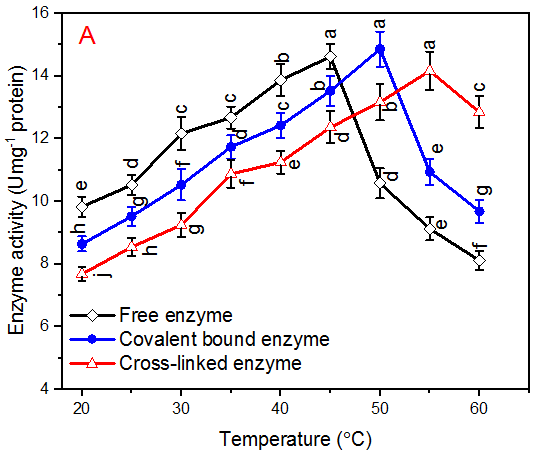

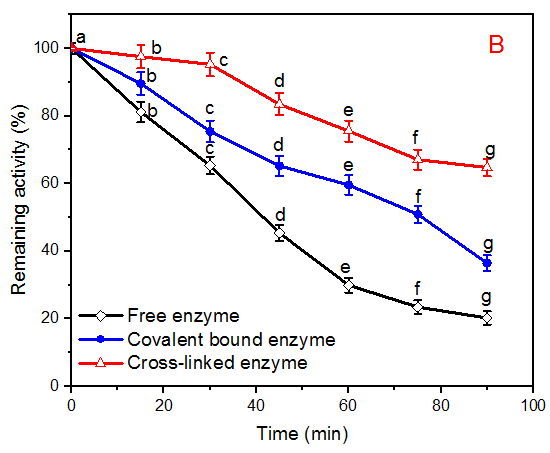


**Supplementary Figure S5. (A) Effect of temperature on free and immobilized β-glucanase from *A. niger*, (B) Thermostability of free and immobilized β-glucanase from *A. niger* at 70^o^C. Enzyme activity was assayed at 40^o^C using CMC as substrate. Vertical bars were displayed as mean ± standard deviation, * p < 0.05, n=3.**

**A**

**C**

**D**

**B**


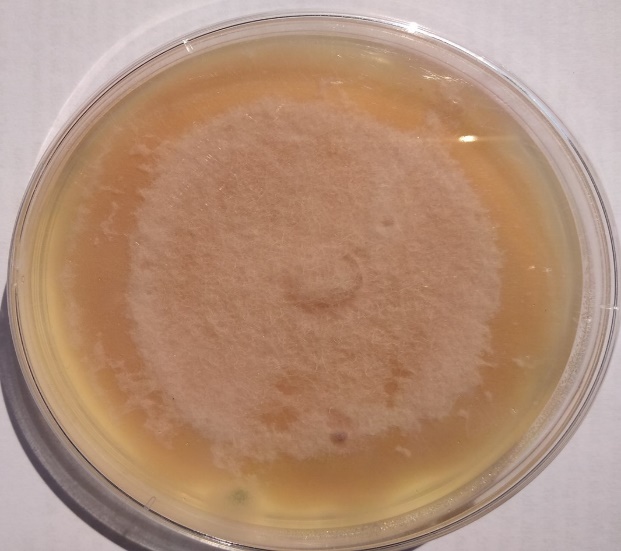

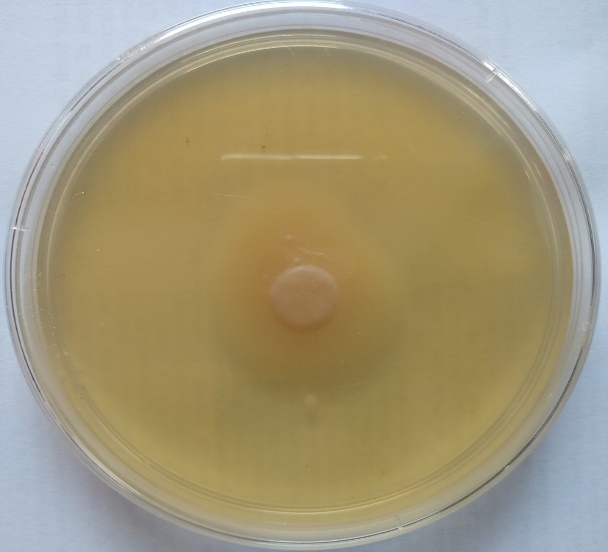

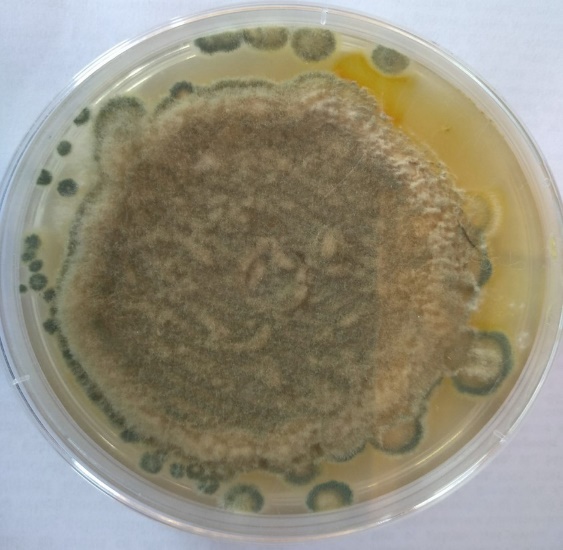

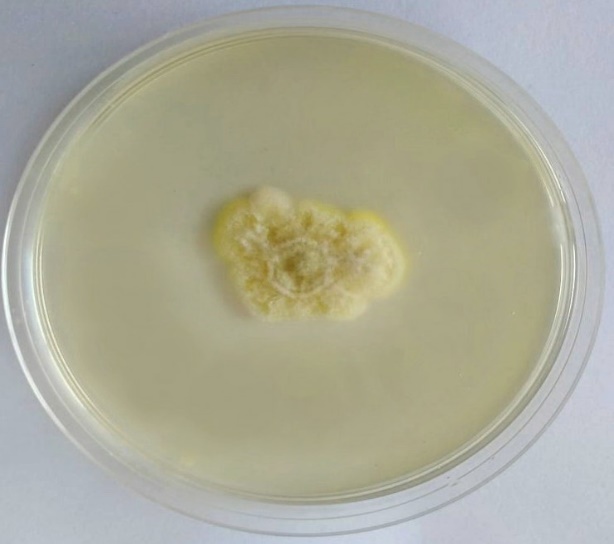


**Supplementary Figure S6. Growth inhibition of *F. oxysporum* (A,C) and *P. digitatum* (B,D) by β-glucanase. The inhibition activity was measured by placing agar disk of fully grown mycelium into SDA medium containing purified β-glucanase: (A-B) negative control and (C-D) purified β-glucanase.**

**Figure S7. Graphical abstract**


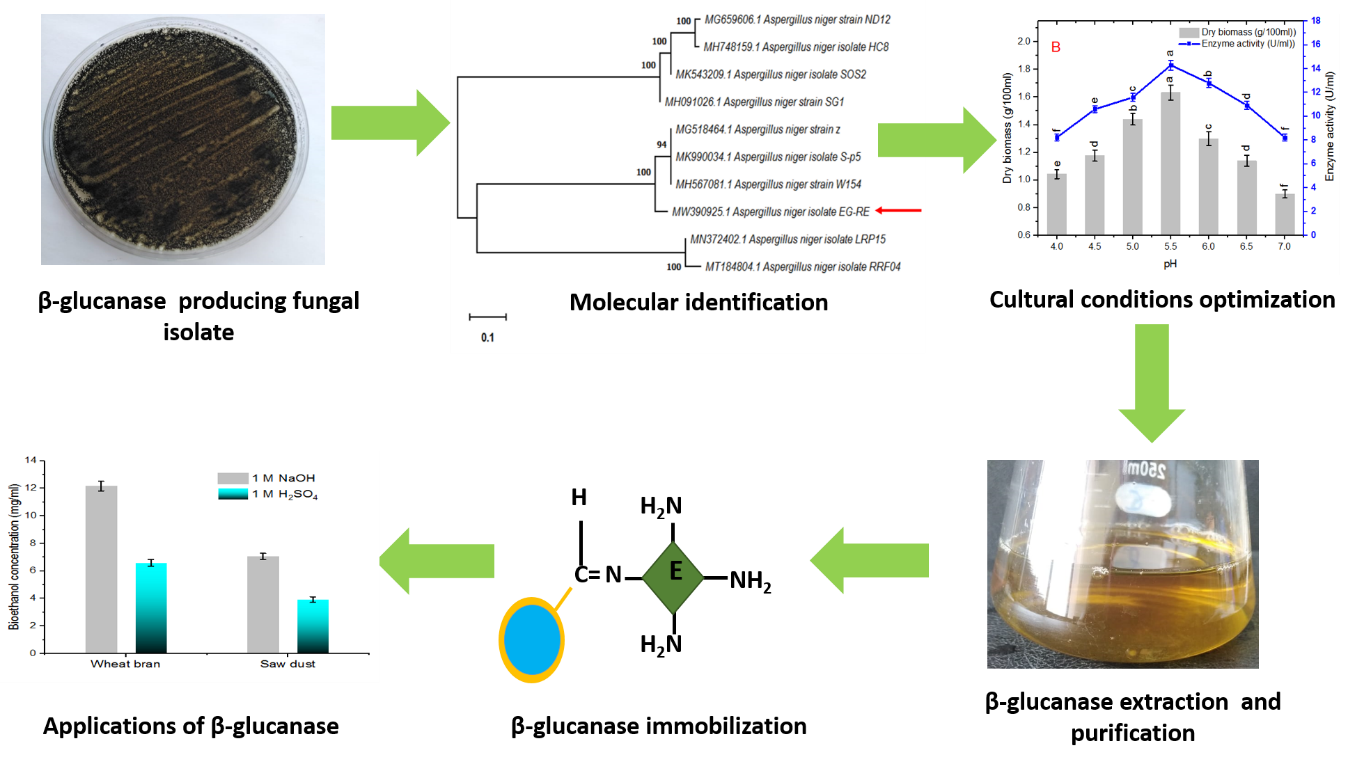

Supplement: Supplementary file 1 — Supplementary Figures. [file 41598_2021_237_MOESM1_ESM.docx]
